# Sudden unexpected death in epilepsy and abnormal glucose metabolism in the rat insular cortex: A brain within the heart

**DOI:** 10.1016/j.clinsp.2022.100059

**Published:** 2022-07-26

**Authors:** Fulvio A. Scorza, Antonio-Carlos G. de Almeida, Carla A. Scorza, Josef Finsterer

**Affiliations:** a Disciplina de Neurociência, Escola Paulista de Medicina, Universidade Federal de São Paulo (EPM-UNIFESP), São Paulo, SP, Brazil; b Centro de Neurociências e Saúde da Mulher “Professor Geraldo Rodrigues de Lima”, Escola Paulista de Medicina, Universidade Federal de São Paulo (EPM-UNIFESP), São Paulo, SP, Brazil; c Laboratório de Neurociência Experimental e Computacional, Departamento de Engenharia de Biossistemas, Universidade Federal de São João del-Rei (UFSJ), São João del-Rei, MG, Brazil; d Klinikum Landstrasse, Messerli Institute, Vienna, Austria

Epilepsy is one of the most common neurological diseases, affects millions of people globally, and available evidence suggests that patients with epilepsy have a higher risk of mortality compared with the general population.^1^^,^^2^ Sudden Unexpected Death in Epilepsy (SUDEP) is the most important direct epilepsy-related cause of death, accounting for 5% to 30% of deaths in individuals with epilepsy, particularly in 20- to 40-year-old patients with refractory epilepsy.^1^, ^2^, ^3^, ^4^ By far, nocturnal Generalized Tonic-Clonic Seizures (GTCS) frequency is the leading risk factor for SUDEP.^1^^,^^2^^,^^5^^,^^6^ Although SUDEP likely does not have a single cause, it is already known that these fatal events are attributable to multiple mechanisms, including cardiac arrhythmias, respiratory dysfunction, and dysregulation of systemic or cerebral circulation.^1^^,^^2^^,^^7^, ^8^, ^9^ This being said, recent efforts to identify brain structural imaging biomarkers of SUDEP have revealed key structures involved in autonomic and respiratory regulation in people who died from SUDEP.^10^, ^11^, ^12^ In these lines, the influence of the insula lobe in SUDEP plays an important role in the current and future scenarios.^13^^,^^14^ The insula is present in all mammals as an integral part of the limbic system, both structurally and functionally.^13^^,^^15^ In fact, as both cardiac and blood pressure regulatory representation show lateralization within the insula of several species, it is clearly established that derangements of insular functions by primary and secondary cerebral insults can significantly affect the cardiac structure, electrophysiology, and contractility and trigger significantly and occasionally fatal cardiac arrhythmias.^15^ Specifically, in epilepsies, translational studies suggest a crucial role of the insula in cerebrogenic cardiovascular disturbances and SUDEP.^12^, ^13^, ^14^^,^^16^ In brief, it has been shown that right or left lesional insular epilepsy may result in ictal bradycardia and asystole and postictal cardiac dysrhythmia.^14^^,^^17^, ^18^, ^19^ Also, recent studies found abnormal autonomic function, characterized by marked differences in Heart Rate Variability (HRV) patterns, in individuals with radiological evidence of insular involvement after epilepsy surgery.^14^ In a general context, as the anatomical substrate of epileptic activity in the CNS manifests a direct relationship with cardiovascular alterations,^20^ it is possible that patients with refractory epilepsy associated and insular lesions are at particular risk of SUDEP. Based on these important clinical findings, the present study's research group used the pilocarpine model of epilepsy, a valuable tool to study the pathophysiology of Temporal Lobe Epilepsy (TLE) in humans,^21^ to evaluate the glucose metabolism in the insula using the 2-[^14^C] deoxyglucose [^14^C-2DG] autoradiographic technique in chronic epileptic rats. The ^14^C-2DG method or “metabolic encephalography”, developed by Sokoloff and colleagues in 1977, provides information concerning functional activity in specific regions of the brain.^22^, ^23^, ^24^ With this in mind, the authors observed in the present study a decrease in ^14^C-2DG labeling (-54%) in the insula of rats with epilepsy as compared with control animals (Fig. 1). Furthermore, cerebral glucose utilization rates measured on labeled insular cortex did not differ among nonepileptic control animals (Fig. 1).

**Fig. 1:**
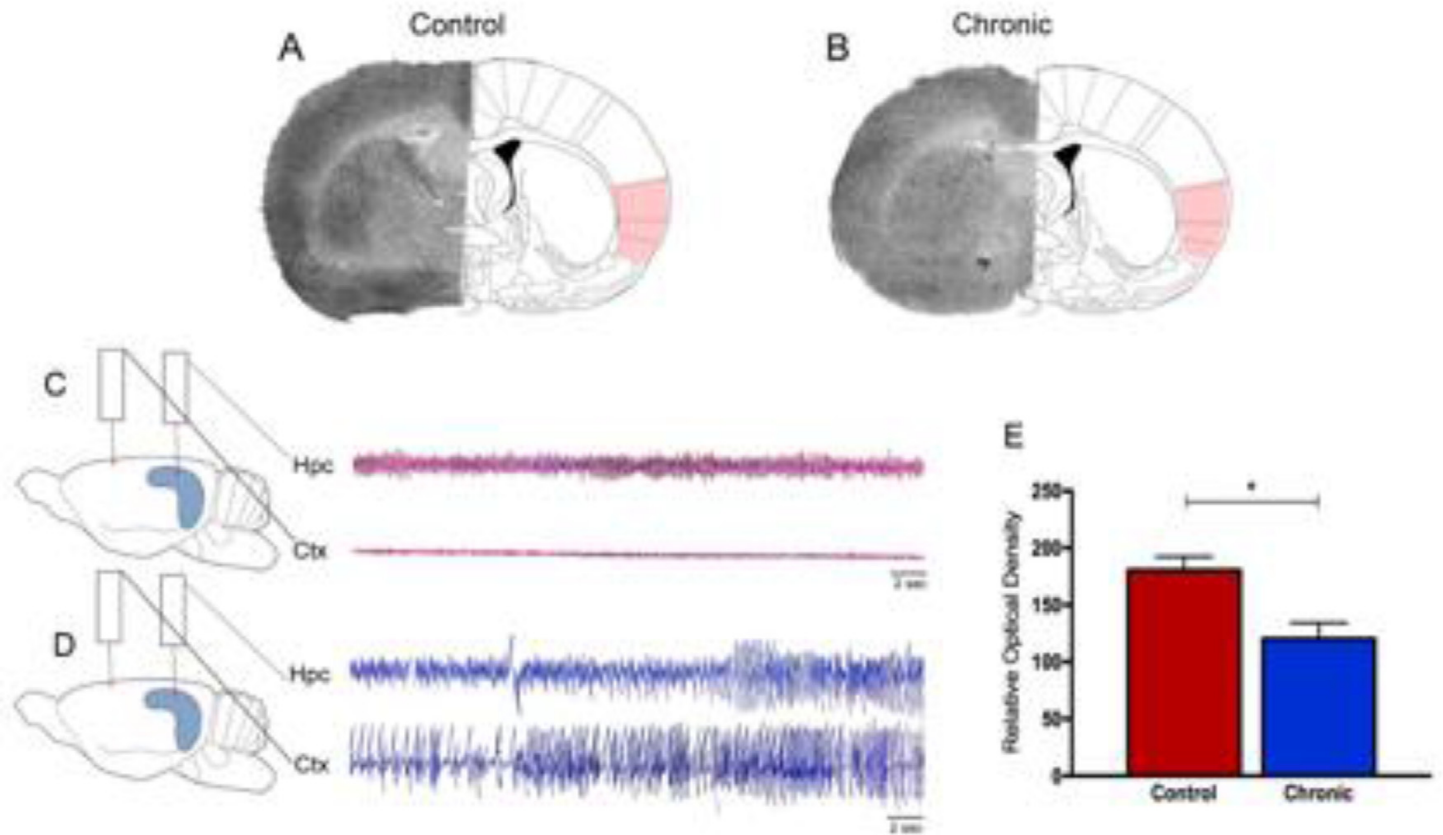
Representative ^14^C-2DG autoradiographs in (A) control rats and (B) rats with epilepsy. Electroencephalograph traces were obtained from control animals (C) and animals with epilepsy (D). Graphic representation of basal levels of cerebral energy metabolism in the control and experimental animals (E).

What do these results tell us? In the last decades, important advances have been achieved in understanding the brain sites involved in cardiovascular control in patients with epilepsy.^10^, ^11^, ^12^, ^13^^,^^16^^,^^20^ In this perspective, the authors are totally in agreement that the insula lobe is probably a key cerebral region involved in SUDEP since abnormalities in this brain structure can lead to cardiac and respiratory dysfunction, central respiratory inhibition, and apnea and arrhythmias in people with refractory epilepsy.^13^ In the authors’ experience, the authors are convinced that the use of experimental models of epilepsy is a valuable tool to investigate the occurrence of SUDEP.^25^ In this sense, current research highlights that hypoventilation, apnea, respiratory distress, pulmonary hypertension, autonomic dysregulation, and arrhythmia are common findings in epilepsy models.^25^ Importantly, the results of this study confirm preliminary data that SUDEP, at least in some cases, could be attributed to dysfunction or morphological alteration of specific brain structures.^26^

Overall, epilepsy is a heterogeneous, multifactorial, and systemic disease. Moreover, epilepsy is a neurological condition with cases of premature death. Finally, neuroscientists should consider that the presence of insular dysfunction in patients with epilepsy could underlie some processes that culminate in SUDEP.

## Conflicts of interest

The authors declare no conflicts of interest.
